# Thin Al_1−*x*_Ga*_x_*As_0.56_Sb_0.44_ diodes with extremely weak temperature dependence of avalanche breakdown

**DOI:** 10.1098/rsos.170071

**Published:** 2017-05-17

**Authors:** Xinxin Zhou, Chee Hing Tan, Shiyong Zhang, Manuel Moreno, Shiyu Xie, Salman Abdullah, Jo Shien Ng

**Affiliations:** Department of Electronic and Electrical Engineering, University of Sheffield, Sheffield S1 3JD, UK

**Keywords:** Al_1−_*_x_*Ga*_x_*As_0.56_Sb_0.44_, avalanche photodiode, temperature coefficient, avalanche breakdown

## Abstract

When using avalanche photodiodes (APDs) in applications, temperature dependence of avalanche breakdown voltage is one of the performance parameters to be considered. Hence, novel materials developed for APDs require dedicated experimental studies. We have carried out such a study on thin Al_1–*x*_Ga*_x_*As_0.56_Sb_0.44_ p–i–n diode wafers (Ga composition from 0 to 0.15), plus measurements of avalanche gain and dark current. Based on data obtained from 77 to 297 K, the alloys Al_1−*x*_Ga*_x_*As_0.56_Sb_0.44_ exhibited weak temperature dependence of avalanche gain and breakdown voltage, with temperature coefficient approximately 0.86–1.08 mV K^−1^, among the lowest values reported for a number of semiconductor materials. Considering no significant tunnelling current was observed at room temperature at typical operating conditions, the alloys Al_1−*x*_Ga*_x_*As_0.56_Sb_0.44_ (Ga from 0 to 0.15) are suitable for InP substrates-based APDs that require excellent temperature stability without high tunnelling current.

## 1. Introduction

Avalanche photodiodes (APDs) are widely used in optical communication, imaging and sensing applications that require detection of high speed and/or weak optical signals. When the noise is dominated by the amplifier, the APD improves the system signal to noise ratio, by amplifying the photo-generated current through the impact ionization process. A cascade of impact ionization events can transform a single electron (or hole) into an avalanche of new carriers, leading to a large external current. In most semiconductor materials, the ionization process is stochastic and hence a mean avalanche gain, *M*, is measured in practice.

Avalanche gain versus reverse bias characteristic, *M*(*V*), of an APD can be highly temperature-sensitive; therefore, a control circuit is essential for accurately adjusting the reverse bias (or the operating temperature) to maintain *M*. When the APD is operated in the Geiger mode, the temperature dependence of avalanche gain can lead to significant changes in the breakdown probability, which in turn governs the photon detection probability and the dark counts. Hence, APDs with temperature-insensitive *M*(*V*) characteristics that require minimal temperature stabilization control are highly desirable.

Temperature sensitivity of an APD is characterized by the temperature coefficient of its avalanche breakdown voltage, *C*_bd_ *=* Δ*V*_bd_*/*Δ*T*, where Δ*V*_bd_ and Δ*T* are changes in breakdown voltage and temperature, respectively. For a given avalanche material, *C*_bd_ increases with avalanche region width, *w* [1,2]. This is illustrated by *C*_bd_ values of 6 and 11 mV K^−1^ reported for InP diodes with *w* = 130 and 250 nm, respectively [1]. Although reducing *w* leads to reduced *C*_bd_, there exists a lower limit for *w*, imposed by the band- to-band tunnelling current from the avalanche region, which is subjected to high electric field. For example, for APDs used in 10 Gbit s^−1^ optical communication receivers, the approximate lower limits for avalanche materials InP and InAlAs are *w* = 180 and 150 nm, respectively [3].

The material AlAs_0.56_Sb_0.44_ (AlAsSb), which is lattice matched to InP substrates, has been investigated as avalanche material for APDs grown on InP substrates [4]. In [4], an AlAsSb diode with *w* = 80 nm exhibited the smallest *C*_bd_ value (0.95 mV K^−1^) reported in the literature for comparable *w* (where the breakdown mechanism is dominated by avalanche breakdown in all data compared). Besides the low *C*_bd_ value, AlAsSb also exhibit very low excess noise factor (*F* = 2.15 at *M* = 10) [5], comparable to that of silicon APD. While exhibiting low excess noise and *C*_bd_ value, the high Al composition is vulnerable to oxidization, which gives higher leakage currents [6]. Incorporating Gallium into AlAsSb was reported to significantly reduce the oxidation rate [7]. More recently, alloys of Al_1−_*_x_*Ga*_x_*As_0.56_Sb_0.44_ (*x* = 0, 0.05, 0.1, 0.15), also lattice-matched to InP substrates, were found to have substantially lower room temperature surface leakage current for alloys with *x* = 0.1 and 0.15 [6]. From AlAs_0.56_Sb_0.44_ to Al_0.85_Ga_0.15_As_0.56_Sb_0.44_, the bandgap reduces very slightly from 1.64 to 1.56 eV [6]. Crucially, the reduced bandgap did not lead to significant band-to-band tunnelling current, suggesting that Al_1−_*_x_*Ga*_x_*As_0.56_Sb_0.44_ could be an attractive material for low tunnelling, low surface leakage avalanche region. Thus, a thin Al_0.85_Ga_0.15_As_0.56_Sb_0.44_ avalanche layer was used to achieve an APD with very high gain–bandwidth product of 424 GHz [8]. For a more comprehensive assessment of Al_1−_*_x_*Ga*_x_*As_0.56_Sb_0.44_ alloys, lattice matched to InP, it is important to consider their temperature sensitivity of dark current and avalanche breakdown, which has not been reported. Note that these alloys have larger bandgap than, and distinct band structures from, the Al_1−_*_x_*Ga*_x_*As_1−_*_y_*Sb*_y_* alloys (*x* = 0.40–0.65 and *y* = 0.035–0.054) grown lattice-matched to GaSb substrates [9].

In this work, we report the temperature dependence of *M*(*V*), from which the *C*_bd_ values for Al_1−_*_x_*Ga*_x_*As_0.56_Sb_0.44_ *p^+^in^+^* diodes, with *x* = 0, 0.05, 0.1 and 0.15, were obtained. The diodes have nominal *w* = 100 nm, a useful value for designing high-speed APDs. Levels of dark currents from Al_1−_*_x_*Ga*_x_*As_0.56_Sb_0.44_ were also compared with those from InP and InAlAs, current avalanche materials in APDs latticed-matched to InP substrates.

## 2. Experimental details

**Figure 1.**
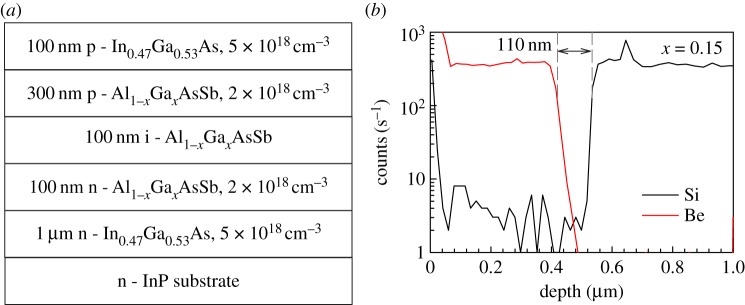
(*a*) Structure details of Al_1−_*_x_*Ga*_x_*As_0.56_Sb_0.44_ wafers and (*b*) secondary-ion-mass spectroscopy data of the Al_0.85_Ga_0.15_AsSb diode.

The Al_1−_*_x_*Ga*_x_*As_0.56_Sb_0.44_ (Al_1−_*_x_*Ga*_x_*AsSb) *p^+^in^+^* diode wafers with *x* = 0, 0.05, 0.1, 0.15 used in this work are identical to those described in [6]. The wafers were grown by molecular beam epitaxy on InP substrate using Be and Si as p-type and n-type dopants, respectively. Structure details of the wafers are shown schematically in figure 1*a*. The wafers were fabricated into circular mesa diodes with nominal diameters, *D* of 400, 200, 100 and 50 µm using standard photolithography and wet chemical etch. The p- and n-metal contacts were formed by Ti–Au.

Capacitance–voltage (*C–V*) measurements on each wafer were performed at room temperature. Fittings to the *C–V* characteristics then gave estimated values of *w* as well as the approximate doping concentrations in the claddings and the i-region [6]. Secondary ion mass spectroscopy data (p- and n-type dopant atoms profiles) from the *x* = 0.15 wafer, as shown in figure 1*b*, support the information obtained from *C–V* characteristics. The deduced *w* values range from 110 to 116 nm, slightly thicker than the nominal 100 nm, as summarized in table 1.

**Table 1.** Avalanche region width, bandgap and deduced *C*_bd_ of the wafers.

material	*w* (nm) [6]	*E*_g_ (eV) [6]	*C*_bd_ (mV/K)
AlAs_0.56_Sb_0.44_	111	1.64	1.07–1.08
Al_0.95_Ga_0.05_As_0.56_Sb_0.44_	116	1.61	1.03–1.05
Al_0.9_Ga_0.1_As_0.56_Sb_0.44_	114	1.59	0.95–0.96
Al_0.85_Ga_0.15_As_0.56_Sb_0.44_	110	1.56	0.86–0.91

Avalanche gain measurements were carried out at temperatures of 77, 150, 200, 250 and 297 K, using a Janis ST-500 low-temperature probe station. The measurements relied on phase-sensitive detection of photocurrent versus reverse bias, *I*_ph_(*V*), so that the photocurrent data were unaffected by background radiation, background noise and diode's dark current. A 542 nm wavelength He–Ne laser light, mechanically chopped at 170 Hz, was used to produce the photocurrent (measured with lock-in amplifier). Dividing *I*_ph_(*V*) with the injected primary photocurrent at low bias gave *M*(*V*). The *V*_bd_ for a given device was then obtained from the horizontal intercept of its 1/*M* versus *V* plot. For a given wafer and temperature, *M*(*V*) were measured from two devices.

To assess uniformity of *V*_bd_ value across a given wafer and to support the *V*_bd_ value derived from *M*(*V*), characteristics of reverse dark current versus reverse bias voltage, *I–V*, was measured from seven devices (three of *D* = 400 µm, two of *D* = 200 µm and two of *D* = 100 µm) from each of the wafers at the same temperature points. These measurements were performed using the low-temperature probe station too, and they confirmed that the breakdown voltage is consistent across a given wafer.

## 3. Results

**Figure 2.**
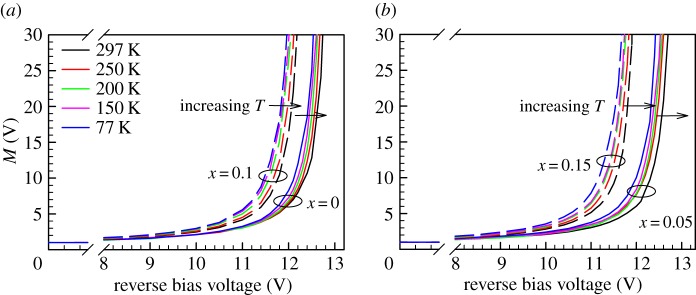
Avalanche gain characteristics from 77 to 297 K of the Al_1−_*_x_*Ga*_x_*AsSb diodes with (*a*) *x* = 0 and 0.10 and (*b*) *x* = 0.05 and 0.15.

**Figure 3.**
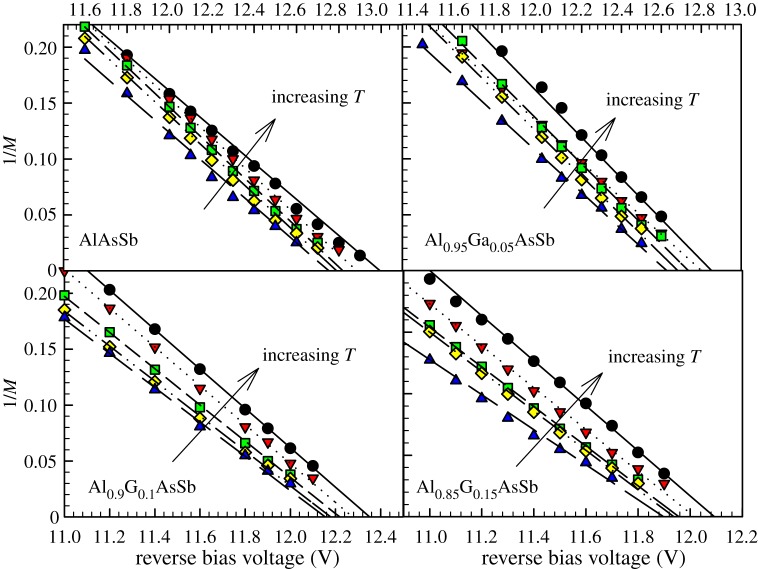
Data of 1/*M* versus reverse bias (symbols) and linear fittings (lines) of the Al_1-*x*_Ga*_x_*AsSb diodes at 77, 150, 200, 250 and 297 K.

The data of *M*(*V*) for all four wafers from 77 to 297 K are shown in figure 2. Results shown were obtained from diodes with *D* = 400 µm. For each alloy, at a given reverse bias, decreasing the temperature increases *M*, which is the typical temperature dependence for avalanche multiplication in most wide bandgap semiconductor materials. Data of 1/*M* versus *V* and linear fittings to extract *V*_bd_ values from one of the devices on all four alloys are shown in figure 3.

**Figure 4.**
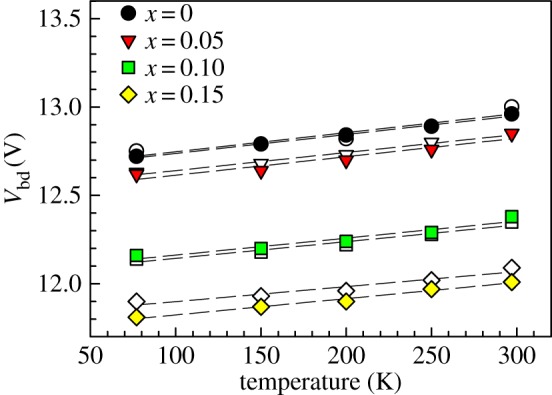
Experimental breakdown voltage versus temperature (symbols) and linear fittings (lines) for the Al_1−_*_x_*Ga*_x_*As_0.56_Sb_0.44_ diode wafers (*x* = 0–0.15). Two sets of data were obtained for each wafer.

The deduced *V*_bd_ values are plotted against temperature for all the alloys in figure 4. The data can be described with linear fittings (with gradients of *C*_bd_) over the range of temperature studied. The values of *C*_bd_ ranging from 0.86 to 1.08 mV K^−1^ are listed in table 1. The *C*_bd_ values for all four alloys are similar, within accuracy of our experimental set-up.

**Figure 5.**
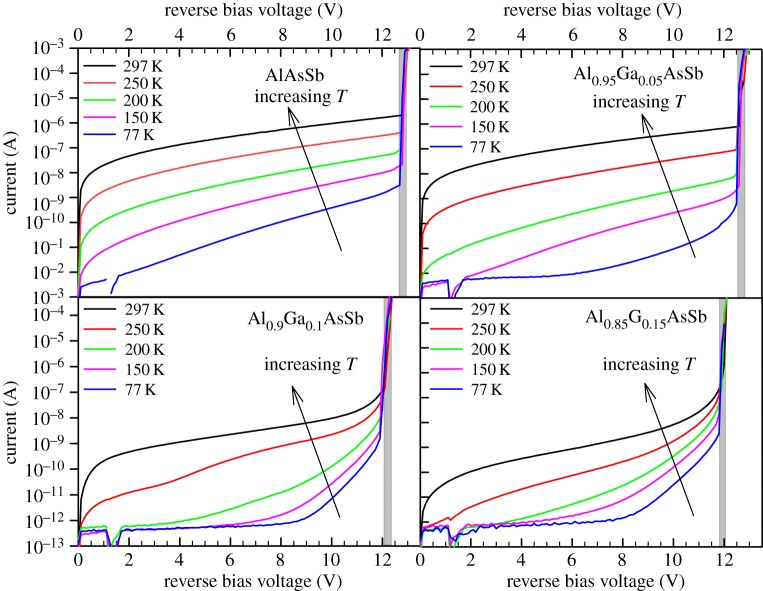
Dark current characteristics (colour lines) and *V*_bd_ deduced from *M*(*V*) data (shaded regions) at 77–297 K of the Al_1−_*_x_*Ga*_x_*AsSb diodes.

The breakdown voltage values deduced using extrapolation of 1/*M* from two devices for each alloy are shown in figure 4. These values are supported by the breakdown voltage obtained from the reverse *I–V* data. For a given wafer, the seven sets of reverse *I–V* data exhibited abrupt breakdown at highly similar voltages, indicating highly uniform diodes. The typical reverse *I–V* data of the *D* = 400 µm diodes at different temperatures are plotted in figure 5 for the four alloys. For each alloy, an abrupt breakdown in dark current can be observed at all temperatures, for reverse bias voltage near the *V*_bd_ deduced from avalanche gain measurements (indicated in figure 5).

Observing figure 5, higher dark currents are present in wafers with higher Al content (smaller *x*). For a given diode, current densities (current divided by device area, not shown here) from different-sized devices at low reverse bias (0 to approx. 8.5 V) showed disagreement, indicating that those dark currents are mainly from surface leakage mechanisms. As temperature falls, these surface leakage currents decrease rapidly, until the measurements of dark current became limited by the measurement system (approx. 0.5 pA).

## 4. Discussion

**Figure 6.**
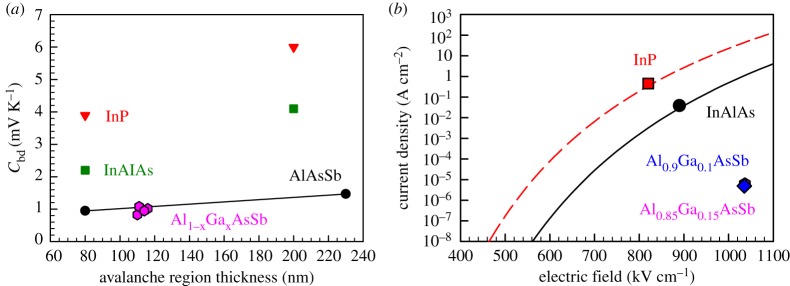
(*a*) Comparison of *C*_bd_ in AlGaAsSb of this work with those for InP, InAlAs [1] and AlAs_0.56_Sb_0.44_ [4]. (*b*) Room temperature comparison of simulated tunnelling current densities for InP [1] and InAlAs [10] diodes with *w* = 110 nm, as well as experimental unmultiplied dark current density of the Al_0.9_Ga_0.1_As_0.56_Sb_0.44_ and Al_0.85_Ga_0.15_As_0.56_Sb_0.44_ diodes at 0.95 *V*_bd_.

In figure 6*a*, the temperature coefficients of breakdown voltage of this work are compared with relevant reports from the literature. Considering only the AlAs_0.56_Sb_0.44_ data from this work and reference [4], *C*_bd_ increases with *w*, as observed on other semiconductors.

The data from this work are also compared with semiconductors used in the avalanche region of InP-based APDs (InP and InAlAs) in figure 6*a*. For a given *w*, the Al_1−_*_x_*Ga*_x_*AsSb diodes have very small *C*_bd_, in line with the *C*_bd_ of AlAs_0.56_Sb_0.44_ diodes from Xie & Tan [4] and much lower than those of InP and InAlAs diodes.

Alloy disorder potential analyses in [11] indicated that the very small *C*_bd_ values for AlAs_0.56_Sb_0.44_ diodes could have originated from significant alloy scattering, because of very different covalent radii of the Sb and As atoms. The ratio between the As and the Sb is the same for the AlAs_0.56_Sb_0.44_ and the Al_1−_*_x_*Ga*_x_*As_0.56_Sb_0.44_ diodes, hence similarly small *C*_bd_ are perhaps not surprising for the Al_1−_*_x_*Ga*_x_*As_0.56_Sb_0.44_ diodes in this work.

The experimental *I−V* characteristics from figure 5 facilitate a comparison of the reverse current density from Al_1−_*_x_*Ga*_x_*AsSb with those from the current avalanche materials of choices, InP and InAlAs. In figure 6*b*, simulated band-to-band tunnelling current density versus electric field from InP [1] and InAlAs [10] *p^+^in^+^* diodes with *w* = 110 nm are plotted, with conditions of 0.95 *V*_bd_ indicated by symbols. Also plotted are the gain-normalized dark current densities at electric fields corresponding to 0.95 *V*_bd_ from our diodes with *x* = 0.10 and 0.15. At conditions of 0.95 *V*_bd_, the dark current densities in our diodes are approximately 5 × 10^−6^ A cm^−2^, at least 5 and 3 orders of magnitude lower than those of InP and InAlAs, respectively. The simulated tunnelling current for thin InAlAs diode is consistent with the level from a waveguide InGaAs/InAlAs APD using an 100 nm thick InAlAs avalanche layer (0.95 *V*_bd_ of 0.096 A cm^−2^) [12]. Our results confirm the potential of using thin AlGaAsSb as a low dark current avalanche region.

## 5. Conclusion

Four Al_1−_*_x_*Ga*_x_*As_0.56_Sb_0.44_ p–i–n diode wafers with 110–116 nm avalanche region width and Ga composition of *x* = 0, 0.05, 0.10 and 0.15 were characterized, in the experimental study on temperature dependence of breakdown voltage, avalanche gain and dark current. All four wafers showed weak temperature dependence of breakdown voltage and avalanche gain, with *C*_bd_ ranging from 0.86 to 1.08 mV K^−1^, among the lowest values ever reported for a wide range of semiconductor materials. Combined with much lower dark current density (compared with tunnelling current densities in InP and InAlAs), the Al_1−_*_x_*Ga*_x_*As_0.56_Sb_0.44_ materials are promising as avalanche layers in InP substrates-based APDs that require excellent temperature insensitivity.

## Data accessibility

The data reported in this article have been uploaded on the Dryad digital repository (http://dx.doi.org/10.5061/dryad.2f1k2) [13].
